# Local and Regional Scale Genetic Variation in the Cape Dune Mole-Rat, *Bathyergus suillus*

**DOI:** 10.1371/journal.pone.0107226

**Published:** 2014-09-17

**Authors:** Jacobus H. Visser, Nigel C. Bennett, Bettine Jansen van Vuuren

**Affiliations:** 1 Department of Zoology, University of Johannesburg, Auckland Park, South Africa; 2 Mammal Research Institute, Department of Zoology and Entomology, University of Pretoria, Pretoria, South Africa; University of Illinois at Urbana-Champaign, United States of America

## Abstract

The distribution of genetic variation is determined through the interaction of life history, morphology and habitat specificity of a species in conjunction with landscape structure. While numerous studies have investigated this interplay of factors in species inhabiting aquatic, riverine, terrestrial, arboreal and saxicolous systems, the fossorial system has remained largely unexplored. In this study we attempt to elucidate the impacts of a subterranean lifestyle coupled with a heterogeneous landscape on genetic partitioning by using a subterranean mammal species, the Cape dune mole-rat (*Bathyergus suillus*), as our model. *Bathyergus suillus* is one of a few mammal species endemic to the Cape Floristic Region (CFR) of the Western Cape of South Africa. Its distribution is fragmented by rivers and mountains; both geographic phenomena that may act as geographical barriers to gene-flow. Using two mitochondrial fragments (cytochrome *b* and control region) as well as nine microsatellite loci, we determined the phylogeographic structure and gene-flow patterns at two different spatial scales (local and regional). Furthermore, we investigated genetic differentiation between populations and applied Bayesian clustering and assignment approaches to our data. Nearly every population formed a genetically unique entity with significant genetic structure evident across geographic barriers such as rivers (Berg, Verlorenvlei, Breede and Gourits Rivers), mountains (Piketberg and Hottentots Holland Mountains) and with geographic distance at both spatial scales. Surprisingly, *B. suillus* was found to be paraphyletic with respect to its sister species, *B. janetta–*a result largely overlooked by previous studies on these taxa. A systematic revision of the genus *Bathyergus* is therefore necessary. This study provides a valuable insight into how the biology, life-history and habitat specificity of animals inhabiting a fossorial system may act in concert with the structure of the surrounding landscape to influence genetic distinctiveness and ultimately speciation.

## Introduction

The life-history of a species coupled with its ecological requirements and vagility determine its dispersal capability [1]. Together with the structure and composition of the surrounding landscape (e.g., barriers to gene-flow) the dispersal capability impacts on the spatial distribution of genetic variation [2]. The interaction of these factors affects genetic structure and species integrities in various taxa inhabiting niches in riverine [3], [4], [5], marine [6], [7], [8], terrestrial [9], [10], arboreal [11], [12], [13] and saxicolous [14], [15], [16], [17] systems. The fossorial/subterranean system, however, remains largely under-investigated. Due to the inconspicuous, subterranean nature of such animals [18], [19], [20], observational methods of population dynamics, breeding system (reproductive interactions) and dispersal behaviour are difficult or not feasible [21], [22], [23], [24], [25], [26], [27], [28].

The structurally simple, constant and predictable subterranean niche [29], [30] has led to specialization (narrower niches) in permanently fossorial taxa [25], [30], [31]. This specialization has resulted in adaptively convergent evolution of both structural and functional traits (low genetic variation, food generalism, cylindrical bodies and anatomical reductions) in fossorial mammals [25], [29], [30], [31]. Consequently, such a specialized morphology decreases vagility [30], [32]. In addition, fossorial mammals, especially rodents, exhibit behavioural attributes (a solitary life-style marked by high territoriality and aggressive behaviour [30], [33]) which acts to limit dispersal. In solitary species, home ranges are synonymous with territories; these defended areas remain fixed for life, with only minor or incremental boundary changes [30], [34], [35], [36], [37] (but see [26] and [28]). As a result, juveniles would have to secure their own territories - a factor influenced by the abundance of the species in the particular habitat. In “saturated” habitats, juveniles would likely traverse larger distances to establish territories. Additionally to these behavioural and biological attributes, suitable habitat patches have a disjunct distribution with restricted food resources [30], [32], [38], [39]. Populations of fossorial mammals are therefore often spatially isolated with patchy distributions [30], [32], [38]. The genetic patterns evident in such a system, according to Nevo [30], should therefore be dominated by isolation-by-distance with low gene-flow between demes (but see [28]), founder effects, genetic drift and inbreeding.

Intuitively, given these life-history characteristics, the landscape in the form of geographic barriers (e.g., mountains and rivers) should also have a profound influence on the distribution of genetic variation in fossorial systems. Although the effect of such geographic barriers on the genetic structure and evolutionary patterns of subterranean species has been widely suggested [30], [32], [34], [40], [41], [42], [43], [44], [45], [46], [47], [48], [49], the effects of these barriers on processes such as gene-flow remain largely speculative.

The family Bathyergidae offers an enticing opportunity to disentangle the factors which influence the spread of genetic variation in fossorial systems. The bathyergids are a monophyletic group of obligatory subterranean hystricognath rodents endemic to sub-Saharan Africa. Six genera are recognized: *Heterocephalus*, *Heliophobius*, *Bathyergus*, *Georychus*, *Cryptomys* and *Fukomys* [27], [31], [40], [41], [44], [50], [51], [52]. Speciation in at least two of the genera is prolific [40] and has been suggested to be linked to population structure and geographic isolation coupled with a labile karyotype [18], [32], [45], [53], [54], [55], [56], [57], factors which result in rapid fixation of e.g., chromosomal mutation [30], [58].

In spite of these suggested models and the possible influence of behaviour, morphology and the landscape on the distribution of genetic variation in fossorial taxa, previous studies focussed mostly on inter-generic relationships [41], [44], [45], [50], [51], [59], [60], [61] or had limited geographic sampling within genera [60]. Few studies have been conducted on geographic variation within either genera or species [41], but more recent investigations have started to disentangle intra-generic relationships (e.g., [31], [41], [43]).

The solitary mole-rat species have been largely neglected in phylogeographic studies compared to their social counterparts [43], [52]. The uncertainty around the intra-generic placement of certain taxa is exemplified by the genus *Bathyergus*, containing two species, *B. janetta* and *B. suillus* [31], [50], [62]. While *B. suillus* and *B. janetta* are proposed to differ in natural history, chromosome number, allozymes and mitochondrial DNA profiles, no genetic differences in either allozymes or karyotype were found by Janecek *et al*. [51] and Deuve *et al*. [58] respectively. Indeed, these species have been suggested to have a hybrid zone at the border of their distributions (Rondawel, South Africa; [31], [58]). As such, any putative genetic differences between these species do not prevent hybridization. In addition, although inter-generic studies have invariably found *B. suillus* and *B. janetta* as sister species [31], [50], [51], [58], [62], [63], these studies used representatives of *B. suillus* from the west coast area, thereby not allowing for the inclusion of possible geographic variation. Ingram *et al*. [41] used representatives of *B. suillus* from both the south- and west coast and reported *B. suillus* to be paraphyletic with respect to *B. janetta*; this led to the suggestion of higher genetic variation in this genus than previously anticipated.

In this study, we use the Cape dune mole-rat, *Bathyergus suillus*, as a model species to investigate the effect of a fossorial life-style on the distribution of genetic variation in a discontinuous landscape divided by barriers. *Bathyergus suillus* is the largest of the mole-rat species, is solitary and highly aggressive and its size (energetic input of digging; [64], [65]) restricts it to the mesic sandy soil areas of the Cape Floristic Region of South Africa characterised by predictable rainfall [27], [31], [37], [51], [65], [66], [67], [68], [69], [70]. The tunnel system of *B. suillus* is relatively short (<400 m; [36]) and the morphology, life-history and behaviour of *B. suillus* lends itself to poor dispersal, thus one would expect genetic structure between isolated populations. Despite this intuitive view, very few studies (with the exception of [28]) have to date focussed on the phylogeography and gene-flow of this species.

We therefore tested hypotheses on how the ecology, distribution, life-history and the connectivity of the surrounding landscape have shaped genetic variation across the distribution of *B. suillus*. Our aims were several fold: 1) to test the model of genetic isolation, inbreeding and diminished heterozygosity proposed by Nevo [30], 2) to investigate the effect of geographic barriers on gene-flow patterns in a fossorial system using *B. suillus* as a model, 3) to determine the phylogeographic patterns and intra-generic relationships within *B. suillus* and 4) to compare our results to previous studies on taxa exhibiting similar life-histories and habitat requirements and interpret the impact of a fossorial life-style on the distribution of genetic variation. By using mitochondrial (cytochrome *b* and control region) and nine nuclear (microsatellite) markers, we determined the distribution of genetic variation at local (Sandveld Bioregion - an area divided by the Verlorenvlei and Berg Rivers as well as the Piketberg Mountains) and regional (Cape Floristic Region- a region cleaved by major river systems and mountain ranges) spatial scales through adopting a landscape genetics approach. The influence of connectivity on spatial genetic patterns is the crux of the field of “landscape genetics” [71]. This emerging field offers a framework by which one can isolate the influence of landscape variables and their impact on genetic variation [71], [72], [73] as well as the identification of barriers to gene flow [74] and integrates ecology, spatial statistics and population genetics to explain evolutionary patterns and processes [71], [73], [75], [76]. Importantly, the movement of an organism is assessed from that organism’s perspective [75]; an organism’s perception of the landscape differs from the simplistic simulations incorporated in isolation-by-distance (IBD) analyses [72]. This study therefore gives insight into understanding spatial and temporal patterns and processes of biotic diversity across different hierarchical levels in a fossorial system.

## Materials and Methods

### Sample collection

**Figure 1:**
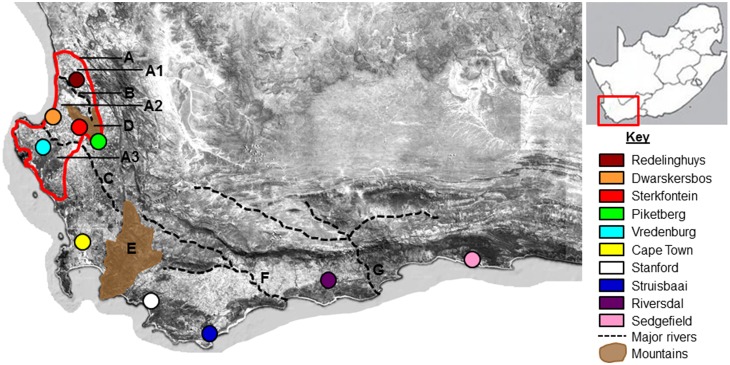
Sampling map of the 10 *B. suillus* populations in this study. Map showing the 10 *B. suillus* collection sites sampled across the Cape Floristic Region of South Africa. The geographic phenomena named in this study are A.) the Sandveld region with A1) the Redelinghuys, A2.) inner-Sandveld and A3.) Vredenburg areas, B.) the Verlorenvlei- and C.) Berg Rivers, D.) the Piketberg mountain, E.) the Hottentots Holland Mountains, F.) the Breede River and G.) the Gourits River. (The sampling map was adapted from ESRI (2007) ArcGIS version 9.3 Media Kit. Redlands, CA: Environmental Systems Research Institute).

Sampling was conducted in a hierarchical fashion across the range of *B. suillus*. Tissue samples were collected from five localities (local scale) in the Sandveld region - an area of Quaternary sand deposits and high species diversity situated between the Atlantic Ocean and the western branch of the Cape Fold Mountains [76], [77]. Specifically, the Sandveld is divided into three regions by the Berg and Verlorenvlei Rivers namely a northern (Redelinghuys), inner (the area between these rivers) and southern (Vredenburg) region. In total, 10 localities (regional scale) were sampled over the species’ range (Figure 1). A total of 20 specimens were sampled per locality with the exception of Stanford (n = 12) and Sedgefield (n = 10). Tail-clippings were taken and stored at room temperature in a saturated salt solution supplemented with 20% dimethyl sulfoxide (DMSO). The protocol was approved by the Ethics Committee of Stellenbosch University (Permit Number: 10NP_VAN01). Handling time was minimized and clipped tails were treated with antibiotics so as to minimize suffering. We are grateful to farmers and landowners in the Western and Southern Cape for access and sample collection (WCNC permit number: AAA-004-00476-0035).

### DNA extraction and sequencing

#### Mitochondrial DNA

Total genomic DNA was extracted from tail-clippings using a commercial DNA extraction kit (DNeasy Tissue and Blood kit; Qiagen) following the manufacturer's protocol. Two mitochondrial DNA segments were amplified using universal primers for the amplifications and sequencing: 928 bp of the protein coding cytochrome b gene (*L14724* and *H15915*; [78], ) and 897 bp on the 5′ side of the hypervariable control region (*LO* and *E3*; [80]).

PCR amplifications followed standard protocols. In short, amplifications were carried out in a GeneAmp PCR 2700 system (Applied Biosystems) at region-specific annealing temperatures (50°C for cytochrome *b* and 48°C for the control region) following the protocols outlined in Karsten *et al*. [81]. Successful amplifications were verified on a 1% agarose gel. Sequencing reactions were performed using the protocols outlined in Jansen van Vuuren and Chown [82]. Electropherograms of the raw data were checked manually (Geneious Pro 5.0 software; Biomatters Ltd, New Zealand) and aligned in MacClade version 4.06 for OS X [83].

#### Microsatellites

Nine microsatellite loci (*DMR1*, *DMR5*, *DMR7*, *CH1*, *Bsuil01*, *Bsuil02*, *Bsuil04*, *Bsuil05*, and *Bsuil06*) were selected from Burland *et al*. [84] and Ingram [43]. These loci were chosen for ease of amplification and polymorphism detected in various populations. The forward primer of each primer pair was 5′-labelled with one of four fluorophores (6-FAM, HEX, VIC and NED). Following primer optimization, all loci were amplified at 60°C; subsequent amplifications were performed in a multiplex at this annealing temperature. For genotyping 1 µl of diluted (1/80) PCR products was combined with 15 µl of deionized formamide and 0.2 µl of the GS500LIZ size standard (Applied Biosystems). Samples were genotyped on an ABI 3170 Prism (Applied Biosystems) and scored using ABI Prism Genemapper version 3.7 software (Applied Biosystems).

### Data analyses

#### Summary statistics and inbreeding

All microsatellite loci across all populations were tested for Hardy-Weinberg equilibrium in Genalex as well as for null alleles in Genepop version 4.0.10 [102], [103]. A test value larger than 0.2 suggests that the marker contains null alleles in a particular population [104]. Linkage disequilibrium was also investigated using Genepop version 4.0.10 [102], [103] by running Markov chains for 10 000 iterations.

Genetic diversity detected within each sampling locality and summary statistics for the combined mitochondrial DNA analyses (including number of haplotypes and nucleotide (π) diversity) were calculated in Arlequin version 3.5 [97]. Similar measures of genetic diversity resulting from the microsatellite analysis were also calculated; these included allelic diversity indices (total number of alleles and mean number of alleles per locus; FSTAT version 2.9.3.2; [105]), observed as well as expected heterozygosities (Genalex version 6.4; [98]). Inbreeding in each colony was assessed by Wright’s F_IS_ (FSTAT version 2.9.3.2).

Fluctuations in population size (based on the combined mitochondrial DNA data) in sampling localities were investigated using Fu’s Fs (a statistic that evaluates population equilibrium; [106]) in DnaSP 5.10.01 [96]. Bottleneck version 1.2.02 [107] was used to investigate whether demographic changes were evident in the history of each sampling locality based on the microsatellite data. This programme measures recent effective population size changes. A two-phased model of mutation was employed (recommended by Luikart *et al*. [108] for microsatellite data) and the Wilcoxon sign-rank test value was applied to assess the probability that an excess of heterozygosity existed at a significant number of loci in a population.

#### Population analyses

To determine whether genetic variation was geographically significantly structured, Φ_ST_ (mitochondrial) and F_ST_ (microsatellites) were calculated between the Redelinghuys-, inner-Sandveld and Vredenburg localities, and between the West- and two South Coast clades. Additionally, pair-wise Φ_ST_ and F_ST_ values were calculated between localities. Significance was determined through 9 999 permutations of the data (Arlequin version 3.5; [97]). Isolation-by-distance was evaluated for the combined mitochondrial DNA dataset using a Mantel test as employed in Arlequin and using Genalex version 6.4 [98] for the microsatellites. Geographical distances for each of the datasets were respectively determined “as the crow flies” (i.e., the shortest and most direct route between localities rather than along mountain ranges) and based on the coordinates of each sampling point.

The spatial location of genetic clusters in the microsatellite data within the studied areas was determined using Bayesian assignment approaches implemented in Geneland version 2.0.10 [99]. This programme determines the spatial location of populations (without prior input) from multi-locus genotypes through the simultaneous analysis of both genetic and geographical data [99]. A Reversible Jump (RJ) Markov Chain Monte Carlo (MCMC) algorithm was applied to estimate the number and location of genetic clusters (K) across the landscape [99]. Geneland also outperforms other spatial genetic clustering programmes when F_ST_ values are high (i.e., when the number of migrants between populations is low) and is efficient at detecting potential contact zones between populations [100]. As allele frequencies were uncorrelated between sampling localities (calculated in Genalex) and gene-flow was expected to be low, the “no admixture” model with “independent/uncorrelated allele frequencies” was selected. Although biologically less relevant to the study species, the “admixture” model with “correlated allele frequencies” was also applied to the data as it is more powerful at detecting subtle population differentiation. We ran 100 000 permutations with a thinning of every 100 trees to search the optimal spatial distribution of markers. Ten chains were run, and the one with the highest likelihood retained.

Gene-flow among sampling localities was estimated in Lamarc version 2.1.6 [101]. Lamarc involves a Markov Chain Monte Carlo coalescent genealogy sampling approach to calculate parameters such as effective population size, growth rate and immigration rate. The programme was run using the Bayesian search strategy under the GTR model for the combined mitochondrial DNA dataset; 10 initial chains were run for 10 000 generations (burnin = 1 000) and 3 final chains of 5×10^6^ generations (burnin = 10 000) completed the analysis. In the case of the microsatellite data, the “Brownian” model was selected and 10 initial chains were run for 10 000 generations (burnin = 1 000); two final chains of 1×10^6^ generations (burnin = 10 000) completed the analysis.

#### Genealogical and molecular dating analyses

Genealogical analyses were conducted to search for phylogeographic patterns and date divergence events. As the control region matrix contained indels (insertions or deletions), these were treated as missing data in all analyses. The trees generated from the cytochrome *b* and control region sequences were congruent and the data were combined into a single sequence for subsequent analyses. Sequences of *B. suillus* were rooted with four *Heterocephalus glaber* (cytochrome b accession numbers: AY 425916.1 and AY 425919.1; control region accession numbers: U 87531.1, U 87534.1 to U 87536.1) and two *Bathyergus janetta* sequences (cytochrome b accession numbers: AY 425916.1 and AY 425919.1; control region accession number: KM 222199) (combined cytochrome *b* and control region) downloaded from GenBank in the parsimony and Bayesian analyses. For molecular dating, only the cytochrome *b* data were used and outgroups included *B. janetta*, *Georychus capensis* (Accession numbers: AF 012243.1 and AY 425920.1), *Cryptomys hottentotus* (Accession numbers: AY 425870.1 and AY 425884.1), *Fukomys* spp. (Accession numbers: EF 043513.1 and EF 043514.1), *Heliophobius argenteocinereus* (Accession number: AY 425937.1), *H. glaber* (Accession numbers: U 87522.1 to U 87525.1), *Hystrix cristata* (Accession numbers: FJ 472577.1 to FJ 472579.1), *Mus caroli* (Accession numbers: AB 033699.1 and AB 109795.1), *Rattus rattus* (Accession numbers: AB 033702.1 and AF 295545.1), *Aplodontia rufa* (Accession numbers: JX 420113.1 and JX 420115.1) and *Xerus inauris* (Accession numbers: AY 452689.1 to AY 452690).

Phylogenetic trees were constructed using parsimony and Bayesian Inference approaches. For this, haplotypes were considered as the OTU. Parsimony analyses were executed in PAUP* version 4.0 [85]. Trees were generated with heuristic searches and TBR branch swapping using 100 random taxon additions. Statistical confidence in nodes was determined through 1 000 bootstrap replicates [86]. Bayesian Inference trees were constructed in MrBayes version 3.2 [87]. The best-fit substitution model (GTR+I+G) was selected through Modeltest version 3.7 [88] by using the Akaike Information Criterion (AIC) [89]. The programme was run for 5×10^6^ generations with sampling every 100 generations. After discarding the first 25% of the trees as burnin, a majority rule consensus tree with posterior probabilities was constructed. Posterior probabilities >0.90 and bootstraps >70% were considered acceptable support [90].

To obtain estimates of times of divergence for various clades, a relaxed molecular clock approach was adopted in BEAST version 1.7 [91]. Two fossil calibration points (as published by [92]) were specified, including the divergence between *Mu*s/*Rattus* (12±1 Mya) and *Scuiridae*/*Aplodontidae* (37 Mya). Runs were continued for 20×10^6^ generations sampling every 1 000 generations (burnin = 2 000). Results were visualized in Tracer version 1.5 [93].

Phylogenetic trees are not always sensitive enough to detect variation and relationships below the species level [88]. In addition, several assumptions underpinning phylogenetic tree construction (such as evolution is strictly bifurcating) are violated. As an alternative, a haplotype network was constructed (under a 95% confidence limit) using TCS version 1.21 [94]; see also [95] for network choice). To determine the amount of genetic divergence among groups identified in the phylogenetic analyses, sequence divergences (uncorrected) were calculated in DnaSP version 5.10.01 [96].

## Results

### Molecular diversity indices

At the local scale, 98 specimens were sequenced (for both the cytochrome band control region fragments characterized by 54 haplotypes; cytochrome b accession numbers: KJ 866510 to KJ 866607; control region accession numbers: KJ866688 to KJ866785) and genotyped (38 alleles; Table S3). Regionally, we obtained sequence (84 haplotypes; cytochrome b accession numbers: KJ 866510 to KJ 866687; control region accession numbers: KJ 866688 to KJ 866865) and microsatellite (68 alleles; Table S3) data for 178 specimens (Table 1). Less than 5% of the microsatellite dataset comprised missing data (Table S1) with a genotyping error of <0.01 incorrect alleles per genotype. No linkage was detected between loci across the sampled distribution and generally loci did not bear signature of null alleles (Table S1). The exclusion of the loci with higher estimates (>0.1) did not significantly influence analyses and therefore all loci were retained. Several loci were not in Hardy Weinberg equilibrium (HWE) over the landscape (Table S2) with all but one population (Piketberg) showing signs of inbreeding (Table 1).

**Table 1:** Genetic diversity of the sampled *B. suillus* populations.

	Mitochondrial DNA					Microsatellites				
Locality	n	Nucleotidediversity	Number ofHaplotypes	Haplotypediversity	Fu’s F	n	Na	He	F_IS_	Bottleneck(p-value)
Redelinghuys	19	0.013	8	0.860±0.054	7.191*	19	7.889±0.676	0.793±0.020	0.225	0.473
Dwarskersbos	20	0.013	15	0.963±0.028	−0.549	20	8.667±0.866	0.781±0.033	0.139	0.199
Sterkfontein	20	0.01	13	0.912±0.046	0.573	20	8.778±0.878	0.802±0.014	0.211	0.251
Piketberg	19	0.003	4	0.380±0.134	4.060*	20	4.000±0.408	0.524±0.082	−0.024	0.381
Vredenburg	20	0.016	14	0.916±0.055	1.022	20	8.556±1.107	0.793±0.024	0.094	0.240
Cape Town	18	0.01	11	0.928±0.040	1.219	20	7.111±1.124	0.659±0.081	0.038	0.466
Stanford	12	0.008	7	0.773±0.128	2.414	10	4.444±0.669	0.535±0.090	0.192	0.130
Struisbaai	20	0.003	14	0.963±0.021	−5.635*	20	2.667±0.471	0.393±0.082	0.050	0.299
Riversdal	20	0.009	12	0.921±0.042	1.239	19	6.000±0.764	0.651±0.085	0.022	0.375
Sedgefield	10	0.009	7	0.867±0.107	1.749	10	3.556±0.709	0.403±0.100	0.106	0.073
**Total**	178	0.043	84	0.979±0.004	1.845	178	6.167±0.336	0.633±0.026	-	-

Genetic diversity for the combined mitochondrial DNA dataset and the microsatellite markers for the 10 *B. suillus* populations sampled across the Cape Floristic Region. In the case of mitochondrial DNA, the number of specimens (n), nucleotide diversity (π), number of haplotypes, haplotype diversity and Fu’s F values in each population is given. Fu’s F values marked with a “*” are significant at p<0.05. For the microsatellites, the number of specimens (n), average number of alleles per population (Na), expected heterozygosity and F_IS_ values within each population are shown together with the test (p) value indicating whether the population experienced a bottleneck during its evolutionary history.

Fu’s Fs values (based on the mitochondrial DNA) indicated that most of the populations were demographically stable (no expansion or contraction); however, the Struisbaai population had experienced a population expansion while the populations of Piketberg and Redelinghuys show signs of population contraction. When the data were pooled, the West Coast (Fu’s F = −12.962; p<0.001) and overall (Fu’s F = −1.845; p<0.05) distributions showed evidence of population expansions while the South Coast region (Fu’s F = 6.245; p<0.01) appears to be demographically contracting. No genetic bottlenecks were evident in the microsatellite data when populations were considered separately (Table 1) or for the combined dataset.

### Population analyses

Significant structure was detected in both datasets at local (Φ_ST_ = 0.35, F_ST_ = 0.13, p<0.001) and regional (Φ_ST_ = 0.82, F_ST_ = 0.099, p<0.001) scales. In addition, significant pairwise differentiation was evident in both datasets between all localities, both at a local and regional scale (Table 2). This genetic structure is likely the result of low (or the absence of) gene-flow between populations (Table 3). Indeed, significant isolation-by-distance was detected over the distribution of *B. suillus* in both datasets on a regional scale (mitochondrial DNA: r^2^ = 0.533; n = 111; p = 0.001, microsatellites: r^2^ = 0.393; n = 178; p = 0.001) and in the microsatellites on a local scale (r^2^ = 0.336; n = 99; p = 0.001). Isolation-by-distance was not found for the mitochondrial data (r^2^ = 0.188; n = 55; p = 0.165) across the Sandveld (local) region.

**Table 2:** Pairwise *ϕ*_ST_/F_ST_ values between the sampled *B. suillus* populations.

Locality	Redelinghuys	Dwarskersbos	Sterkfontein	Piketberg	Vredenburg	Cape Town	Stanford	Struisbaai	Riversdal	Sedgefield
Redelinghuys	/	0.049	0.051	0.220	0.048	0.168	0.252	0.316	0.162	0.285
Dwarskersbos	0.372	/	0.056	0.230	0.055	0.165	0.254	0.318	0.159	0.272
Sterkfontein	0.419	0.098	/	0.231	0.051	0.163	0.241	0.309	0.139	0.253
Piketberg	0.616	0.249	0.307	/	0.247	0.321	0.414	0.450	0.346	0.443
Vredenburg	0.369	0.246	0.299	0.374	/	0.163	0.249	0.316	0.156	0.282
Cape Town	0.675	0.646	0.691	0.786	0.586	/	0.182	0.383	0.192	0.322
Stanford	0.707	0.676	0.719	0.863	0.601	0.497	/	0.517	0.335	0.454
Struisbaai	0.901	0.895	0.908	0.963	0.862	0.916	0.949	/	0.299	0.433
Riversdal	0.871	0.864	0.879	0.937	0.827	0.889	0.922	0.935	/	0.213
Sedgefield	0.855	0.847	0.867	0.944	0.780	0.876	0.919	0.938	0.741	/

Pairwise *ϕ*_ST_/F_ST_ values between the 10 *B. suillus* populations sampled across the Cape Floristic Region. Values above the diagonal are based on the microsatellite (F_ST_) data and those below the diagonal represent the combined mitochondrial sequence data (*ϕ*_ST_). All values were significant at p<0.001.

**Table 3:** Gene-flow values (number of individuals per generation) between the sampled *B. suillus* populations.

Sampling localities		Mitochondrial DNA		Microsatellites	
		––––––>	<––––––	––––––>	<––––––
Dwarskersbos	Redelinghuys	0.883+1.553–0.813*	1.588+1.938–1.335*	0.593+0.304–0.289	0.172+0.220–0.010
Sterkfontein	Dwarskersbos	1.111+2.217–0.997*	1.221+1.544–1.186*	1.739+0.003–0.653*	1.859+0.051–0.045*
Vredenburg	Dwarskersbos	0.002+0.585–0.002	0.388+1.090–0.387*	1.363+0.055–0.059*	1.108+0.048–0.044*
Piketberg	Sterkfontein	0.295+0.996–0.295*	0.108+0.407–0.107	0.434+0.039–0.041	0.424+0.073–0.041
Cape Town	Vredenburg	0.002+0.314–0.002	0.003+0.318–0.002	0.190+0.393–0.063	0.472+0.002–0.001
Stanford	Cape Town	0.196+1.127–0.195*	0.002+0.781–0.002	0.407+0.006–0.268	1.276+0.067–0.037*
Struisbaai	Stanford	0.001+0.510–0.001	0.000+0.187–0.000	0.063+0.120–0.039	0.243+0.127–0.101
Riversdal	Struisbaai	0.009+0.172–0.009	0.013+0.272–0.013	0.398+0.007–0.024	0.503+0.044–0.035
Sedgefield	Riversdal	0.072+0.672–0.071	0.095+0.506–0.095	0.271+0.146–0.248	0.441+0.058–0.072

Gene-flow between the 10 *B. suillus* populations sampled in the Cape Floristic Region. Calculations are based on both mitochondrial DNA and microsatellite data. Values marked with a “*” are gene-flow levels >1 individual per generation (standard error included).

There was also a significant partitioning of variance in both datasets (Φ_ST_ = 0.49, F_ST_ = 0.06, p<0.001) when the populations were grouped (*a-priori*) into two respective groupings, pertaining to the West Coast (Redelinghuys, Dwarskersbos, Sterkfontein, Piketberg, Vredenburg, Cape Town) and South Coast (Stanford, Struisbaai, Riversdal, Sedgefield). A similar pattern was found locally in the Sandveld (mitochondrial DNA: Φ_ST_ = 0.363, F_ST_ = 0.503, p<0.001) when the populations were grouped into the three respective areas constituting this region (Vredenburg, inner-Sandveld and Redelinghuys).

### Genealogical analyses - affiliation of *B. janetta*

**Figure 2:**
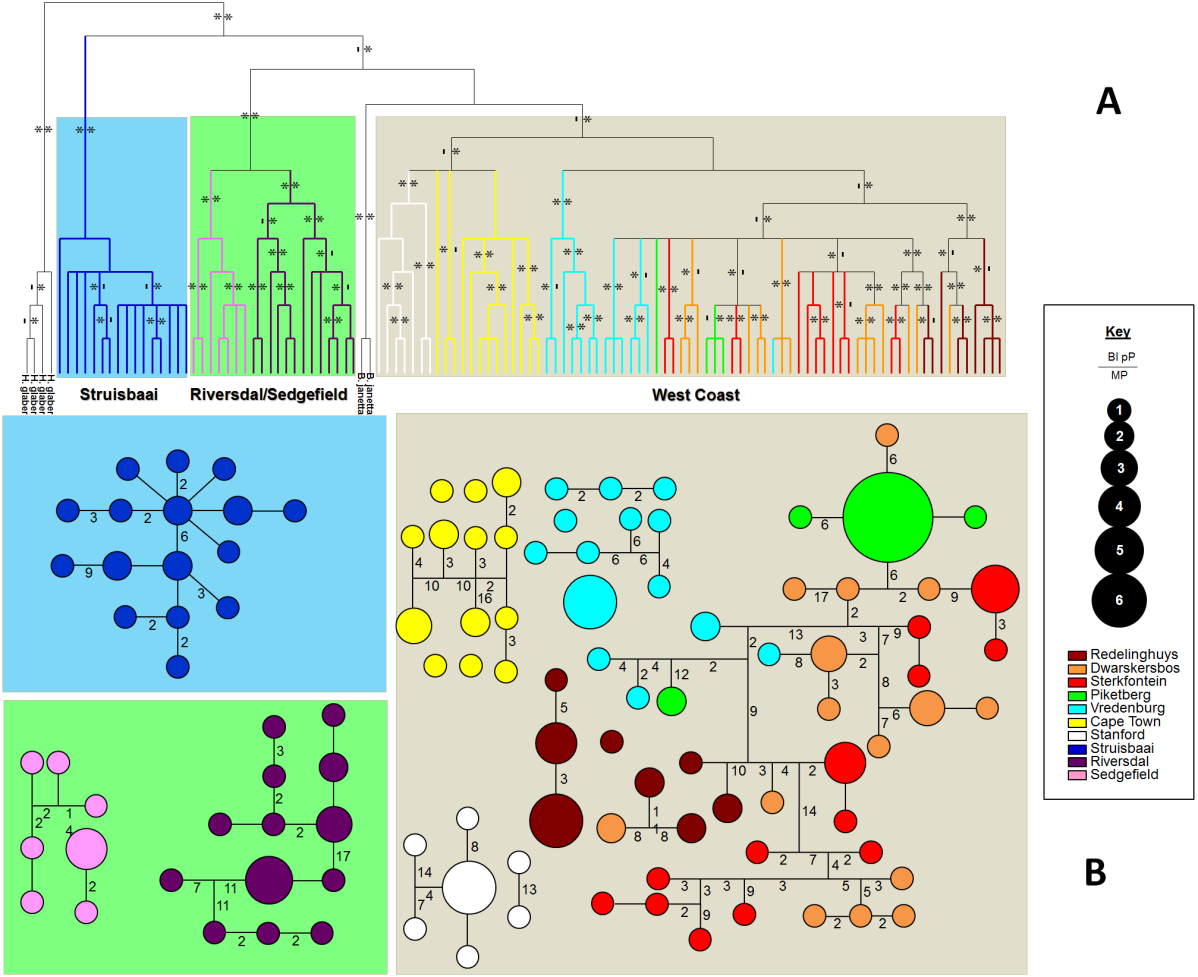
Bayesian phylogram and haplotype network demonstrating the different mitochondrial DNA clades across the sampled distribution. Figure 2A) Bayesian phylogram and B) haplotype networks obtained from the analyses based on combined cytochrome b and control region sequences demonstrating the different mitochondrial DNA clades detected in *B. Suillus* from localities across the Cape Floristic Region. For the Bayesian tree, a “*” above each node represent an acceptable posterior probability (pP>0.90) value derived from the Bayesian inference (MrBayes and BEAST) analyses and those below nodes are the Maximum Parsimony values (MP>70). A “-” indicate that the grouping was not found by the particular analysis. For the haplotype network, the size of each circle reflects the number of specimens with a particular haplotype. Numbers on branches represent the mutational steps separating haplotypes.

*Bathyergus suillus* was found to be paraphyletic with respect to its sister species *Bathyergus janetta*; the latter grouping as a sister taxon (albeit not with strong statistical support in the MP/Bayesian analyses) to the West Coast clade (Figure 2A; Figure S1). Sequence divergence estimates (based on the cytochrome b fragment) between the monophyletic clades are found in Table 4.

**Table 4:** Uncorrected sequence divergence between the clades retrieved in the genealogical analyses.

Clade A	Clade B	Uncorrected SequenceDivergence (%)
West Coast	*B. janetta*	3.9
West Coast	South Coast	3.9
West Coast	Struisbaai	3.8
Sandveld	Cape Town/Stanford	1.3
Struisbaai	Riversdal/Sedgefield	3.2
Struisbaai	Riversdal	3.3
Riversdal	Sedgefield	0.8

Uncorrected sequence divergence estimates (based on the cytochrome b marker) between the different genealogical clades retrieved within *B. suillus* across its distribution.

### Phylogeographic patterns

The trees generated by the various methods were largely congruent with three major clades evident on a regional scale pertaining to the West Coast (including Stanford on the South Coast), Struisbaai and Riversdal/Sedgefield sampling areas respectively (Figure 2A; Figure S1). These three clades are respectively separated by 3.8% (West Coast and Struisbaai) and 3.2% (Struisbaai and Riverdal/Sedgefield) uncorrected sequence divergence for the 928 bp of cytochrome b sequence data. At least four major genetic provinces were evident regionally pertaining to the Sandveld, Cape Town/Stanford, Struisbaai and Riversdal/Sedgefield areas. Of these, the Cape Town/Stanford subclade and Riversdal/Sedgefield clade could further be genetically divided into their constituent populations (Figure 2A; see Table 4 for sequence divergence estimates). On a local scale, there was a signal of three clades (inner-Sandveld, Vredenburg and Redelinghuys) pertaining to the sampled areas on the different sides of rivers. In the inner-Sandveld clade, taxa from different localities were largely paraphyletic. There was, however, an indication of Piketberg- and Sterkfontein subclades within the larger inner-Sandveld clade.

The haplotype network retrieved nineteen haploclades which could not be connected at the 95% confidence level (Figure 2B). There were no shared haplotypes between populations with nearly every population forming a separate haploclade (except those of the inner-Sandveld). Eight haploclades were evident at a local scale that largely consisted of individuals from the Vredenburg, Redelinghuys and the inner-Sandveld populations clustering separately.

### Clustering analyses

**Figure 3:**
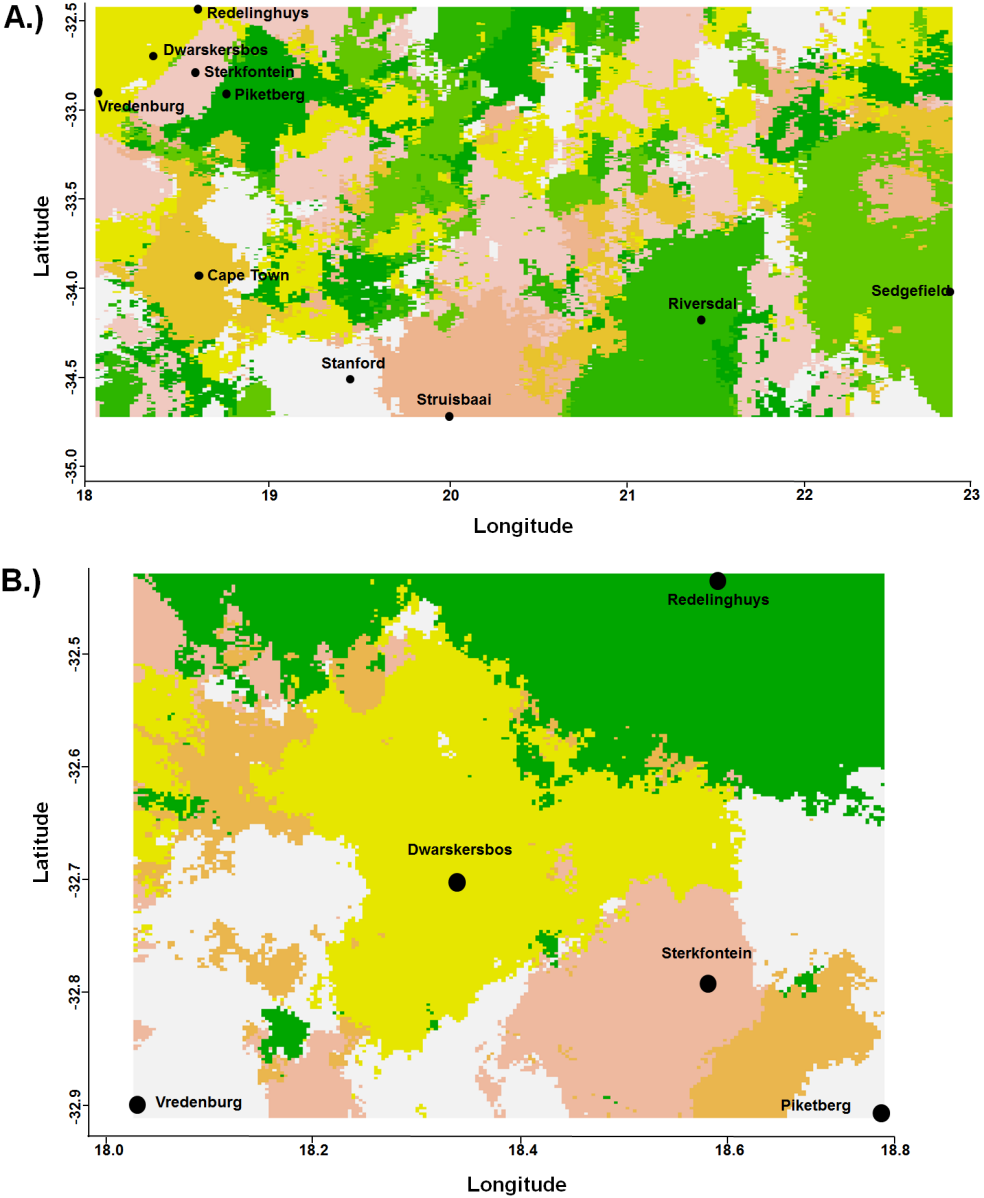
Genetic groupings revealed by the Geneland analysis at regional and local scales. Genetic groupings revealed by the Geneland analysis of the microsatellite data on a A) regional (Cape Floristic Region) and B) local (Sandveld) spatial scale. Dots reflect the location of each population and the colours correspond to each separate genetic grouping.

Invoking the “Admixture” model revealed ten clusters across the landscape thereby confirming the genetic distinctiveness of each sampled population (results not shown). Under the “no admixture” model eight clusters (based on the microsatellite data) were retrieved overall with virtually every sampled population clustering separately (Figure 3A) except for the Redelinghuys/Dwarskersbos/Vredenburg cluster. On a local scale, the aforementioned cluster could be divided into its constituent populations - five populations were revealed across the landscape by both the “admixture” and “no admixture” models (Figure 3B).

## Discussion

### Genetic patterns

Phylogeography of subterranean taxa is characterised by high inter-population differentiation [20], [31], [32], [45], [47], [50], [51] and *B. suillus* is no exception. Genetic diversity is structured between *B. suillus* populations across both local and regional spatial scales (Table 2; also see [28]) with every sampled locality forming a unique genetic entity (Figures 2 and 3).

Such strong genetic sub-structuring points to the isolated nature of populations of fossorial species. Specifically, the isolated nature of *B. suillus* populations (significant isolation-by-distance based on both datasets) may be linked to habitat fragmentation coupled to low vagility; pertinent factors to the genetic sub-structuring of subterranean taxa [32], [34], [39], [109]. The low vagility of *B. suillus* was previously documented by Bray *et al*. [27] (but see [28]) who found the maximum distance of male gene-flow between two populations to be 2 149 m, resulting in low but significant levels of differentiation (Fst = 0.018). Similarly, Bray *et al*. [28] found low but significant differentiation (Fst = 0.02 and Fst = 0.17) across a one kilometre distance in the Cape Town and Darling areas respectively. Not surprisingly, gene-flow between most populations in the present study was effectively negligibly small (Table 3), although low historical genetic exchange was evident on a local scale. Given the separate genetic profiles of each sampled population (Figures 2 and 3; Table 2), it is likely that a.) these estimates are slightly above the realistic value of genetic exchange in the natural system or b.) if these estimates are indeed correct, the genetic exchange is insufficient to homogenize the genetic profiles of populations. It seems likely that gene-flow over longer distances requires stochastic above-ground movement [27],[28],[110], therefore the associated cost of such movement results in genetic structure even at fine spatial scales in fossorial taxa (see also [32] and [110]). Genetic structure and differentiation between fossorial populations may therefore occur even in the absence of geographic barriers.

Although we did not directly estimate effective population sizes, inbreeding (F_IS_) was evident within all but one of the populations (Table 1; also see [28]). Taken together with the gene-flow data (Table 3), the occurrence of non-random mating within populations is likely due to possible small effective population sizes. Genetic sub-structuring may therefore be enforced by a fast accumulation of mutations making genetic drift a pertinent factor in fossorial systems. Accelerated evolution of the mitochondrial genome [50] and specifically the cytochrome b region [42], [111] coupled with a short generation time (one year in *B. suillus*; [30]) likely promotes relatively fast genetic differentiation between isolated *B. suillus* populations.

Environmental heterogeneity is low within the subterranean niche, and therefore homoselection was proposed to drive low heterozygosity in fossorial taxa [29], [30], [50], [112], [113]. Selection-migration theory also impinges on the genetic population structure of fossorial taxa, hence a negative correlation exists between polymorphism and adult mobility [112]. Low heterozygosity has been demonstrated in a variety of subterranean taxa [30], [55], however, these values were based on allozyme data. Levels of expected heterozygosity in *B. suillus* (Table 1) were consequently higher than proposed in the former studies, but were in line with more recent studies which similarly included microsatellite data ([27], [28], Konvičková, 2013, unpublished data). A revision and validation of the earlier model by Nevo [30] by using the more variable markers available is therefore necessary. In addition, the model of small founder populations was only supported in two populations (based on the mitochondrial DNA) whilst most populations were stable (Table 1) or even expanding. The demographic decline observed across the South Coast distribution is likely due to agricultural activities which reduces and fragments available habitat.

### Barriers to gene-flow

The isolated nature of fossorial populations results in genetic structure. It is therefore intuitive to expect that geographic phenomena may act to further fragment such populations and enforce genetic distinctiveness. As such, both mountains and rivers limit genetic exchange between fossorial taxa, including *B. suillus*.

Mountains act as phylogeographic disruptors between *B. suillus* populations at both local and regional scales. On a local scale, the Piketberg Mountain creates a barrier to gene-flow between the Piketberg and inner-Sandveld populations (Figures 2 and 3). Similarly, the Hottentots Holland Mountains act as a barrier to gene-flow separating two of the major clades (West Coast and Struisbaai; Figures 2 and 3; also see [28]) on a regional scale. Mountains, rifting and volcanism are the drivers of differentiation between populations and speciation within the African Bathyergidae, impacting on genetic structure either directly (as geographic barriers; [31], [40], [46]) or indirectly (influencing rainfall patterns and therefore vegetation; [31], [48]).

When specifically looking at phylogeographic patterns, the African Rift Valley (volcanic uplands and deep valleys) divides the distribution range of the most diverse taxa within the Bathyergidae [40], [46] and has influenced the adaptive radiation (speciation) of taxa such as *Fukomys* [46], *Heliophobius* [43], [46], [69] and *Cryptomys* through geomorphological processes [31], [41], [46]. While the early divergences within the Bathyergidae were independent of rifting, later divergences, patterns and distributions were mainly influenced by this geological process [31], [44]. When more local patterns are taken into account, chromosomal variation in *Thomomys bottae* is maximal in insular montane populations, but minimal in plains populations [30]. In line with this, Patzenhauerová *et al*. [110] also reported that barriers (such as a rocky hill) impeded gene-flow between populations of another mole-rat species, the silvery mole-rat, *Heliophobius argenteocinereus*.

Interestingly, the Cape Town and Stanford areas (that span the Hottentots Holland Mountains) group together in the genealogical analyses (Figure 2A). This grouping may be attributed to a colonization event subsequent to the divergence of the West and South Coast clades (30.65+13.93–10.69 Mya; Figure S2). According to Siesser and Dingle [114], a marine regression occurred during this period (∼25 Mya) which would have opened up a large region of the coastal belt (500–600 m) thereby allowing dispersal and establishment of individuals from the Cape Town area in Stanford. Similar colonization events during major marine regressions have been recorded in *Spalax* and *Nannospalax* [48]. When the sequences of Bray *et al*. [28] (Accession numbers: KC 153980 to KC 153987) were added to our cytochrome b dataset, haplotypes 6 and 8 from the Bray *et al*. [28] study (presumable from the Darling area) grouped together with our Sandveld clade while the rest (haplotypes 1 to 5 and 7) grouped with the Cape Town individuals in the genealogical analyses (results not shown). This genetic discontinuity, also documented by [28], shows historic isolation of these areas across the Cape Flats around 12.32+7.36–5.45 Mya - a period characterized by a major transgression phase (15-4 Mya with the sealevel rising to over 300 m above the current level; [114]).

Drainage systems also act as phylogeographic disruptors in *B. Suillus* at both local and regional scales. On a local scale, the Berg- and Verlorenvlei Rivers form phylogeographic barriers to gene-flow thereby structuring genetic variation (Figure 2; also see [47], [49]). Historically, a northward colonization (from the Vredenburg area) of the inner-Sandveld and Redelinghuys areas is evident across these rivers (Figure 2A), therefore stochastic gene-flow events across rivers seems possible. *Bathyergus suillus* prefers lowland sandy areas, especially coastal dunes. These animals therefore occur relatively near the coast and populations would be separated by river mouths (rather than the upper reaches) for most of the time. It is possible that gene-flow and colonization of areas may occur around the upper reaches of these rivers during dry periods (also see [115]). It should be noted that differentiation happened in relative isolation since such colonization events. For instance, the Redelinghuys and Vredenburg haplotypes found to cluster with those of the inner-Sandveld may be attributed to relatively recent colonization of these areas from the same ancestral stock. Therefore, divergence times have been too short to establish monophyly of haplotypes in these areas although distinct genetic profiles are evident.

In contrast, patterns on a regional scale show historic isolation across the larger perennial rivers. The Struisbaai- and Riversdal/Sedgefield clades of the South Coast (Figure 2A; Figure S1) are separated by the Breede River. Similarly, the Riversdal/Sedgefield clade can further be divided into its constituent populations (Figure 2A; Figure S1) showing the action of the Gourits River as a barrier to gene-flow. Indeed, water bodies such as lakes [31], [46] and rivers [32], [42], [43], [45], [47], [49] have impacted on the isolation of fossorial taxa and drainage areas seem to be a hotbed of differentiation due to fragmentation [42], [116]. Specifically, genetic structure in mole-rat populations and speciation due to river barriers has been reported within the Bathyergidae e.g., speciation and karyotypic divergence in *Fukomys* [42], [45], *Cryptomys* [40], [41], [42], [43] and the separation of *Coetomys* from *Cryptomys* [41], [43].

### Taxonomic implications

While *B. suillus* and *B. janetta* are definitive species [31], [50], [51], [58], [62], [63], other studies found contrasting results [41], [51], [58]. Intergeneric studies that found *B. suillus* and *B. janetta* as sister species invariably included sampling bias (e.g., [31], [50], [51], [58], [62], [63]). This was, in a sense, rectified by Ingram *et al*. [41] who found *B. suillus* as paraphyletic with respect to *B. janetta* - the latter also forming a sister taxon to the West Coast group. Similar results were obtained in this study (Figure 2A). A dispersal event of animals from the southern West Coast northward into Namaqualand and Namibia across the Knersvlakte region (via the coastal belt) is therefore the most plausible scenario. The Knersvlakte is a known barrier to gene-flow to even more mobile taxa [15], [16], [117], [118] and divergence between *B. suillus* and *B. janetta* could therefore have occurred in allopatry.

With respect to the genetic structure found within *B. suillus*, the MP and BEAST analyses, indicate that the West Coast clade may be more derived (not found in the Bayesian analysis), suggesting a possible colonization of the West Coast from the South Coast (also see [41], [43]). As further evidence, the sister genus to *Bathyergus*, *Georychus*, has a current distribution along the South Coast of South Africa [67]. The most probable scenario thus entails that *B. suillus* colonized the West Coast after the *Bathyergus*/*Georychus* clade split on the South Coast. This colonization event probably took place during a marine regression in the Miocene [115], [119], [120] via the coastal belt. The various clades within *B. suillus* show very different evolutionary histories. Additionally, the amount of sequence divergence between *B. Suillus* clades (Table 3) is above the inter-specific threshold (based on cytochrome b) reported for mammalian [111] and fossorial taxa [30]. These findings suggest that the genus *Bathyergus* is in need of a taxonomic revision.

## Conclusion

This study exemplifies a holistic approach to investigating the genetic structure within species. The life-history, biology and habitat requirements of fossorial taxa impact on the distribution and isolation of populations - here demonstrated by interpreting phylogeographic and gene-flow patterns in *B. suillus* in the context of previously published biological and ecological information on this species. The model proposed by Nevo [30] to explain the factors (low gene-flow, isolation-by-distance, genetic drift) impacting on the genetic structure of fossorial populations was largely supported by our data; however we could provide no convincing evidence of small founder populations; the comparability of heterozygosity values was compromised by the markers used in the above-mentioned study. Due to the relatively quick generation time of *B. suillus* (a characteristic shared by all rodent taxa), differentiation of isolated populations may happen in a comparatively short period of time. Additionally, geographic barriers to gene-flow (mountains and drainage systems) and geographic distance play a significant role in structuring genetic variation within *B. suillus* - a factor which may partly explain the staggering radiation in fossorial species worldwide. Furthermore, a systematic revision of the genus *Bathyergus* is necessary, given the findings of this study.

## Supporting Information

Figure S1 **Bayesian phylogram demonstrating the different mitochondrial DNA clades across the sampled distribution.** Bayesian phylogram obtained from the analyses based on combined cytochrome b and control region sequences demonstrating the different mitochondrial DNA clades detected in *B. suillus* from localities across the Cape Floristic Region. Values above each node represent posterior probabilities (Pp) derived from the Bayesian inference (MrBayes and BEAST) analyses and those below nodes are the Maximum Parsimony values.
(TIF)

Figure S2 **Bayesian phylogram from the BEAST analysis indicating the divergence dates between clades.** Bayesian phylogram obtained from the BEAST analyses of the cytochrome b haplotypes among the 10 *B*. *suillus* sample sites across the Cape Florisitc Region. The values above each node represent the posterior probability (pP) values derived from the Bayesian inference analyses. The populations comprising the two clades evident across the distribution are shown. The divergence dates for four nodes (A–D) are indicated as bars which include the span of the divergence estimate for that particular node.
(TIF)

Table S1 **Summary information for the microsatellite loci used in this study.** Locus summary information showing the estimated proportion of null alleles (Null), percentage missing data of the total 356 alleles/locus, proportion of missing alleles/locus, the genotyping error per genotype and F_IS_ values for each of the microsatellite loci used in this study.
(DOCX)

Table S2 **Summary of loci which did not conform to Hardy-Weinberg Equilibrium in each population.** Summary of loci which did not conform to Hardy-Weinberg Equilibrium (marked by an “x”) in each sampled population of *B. suillus*.
(DOCX)

Table S3 **Microsatellite data used in this study.**
(XLSX)
