# Supplementary figures and images for: Local and Regional Scale Genetic Variation in the Cape Dune Mole-Rat, Bathyergus suillus
Source: PLoS One. 2014 Sep 17;9(9):e107226. doi: 10.1371/journal.pone.0107226 (PMC4167993; doi:10.1371/journal.pone.0107226)

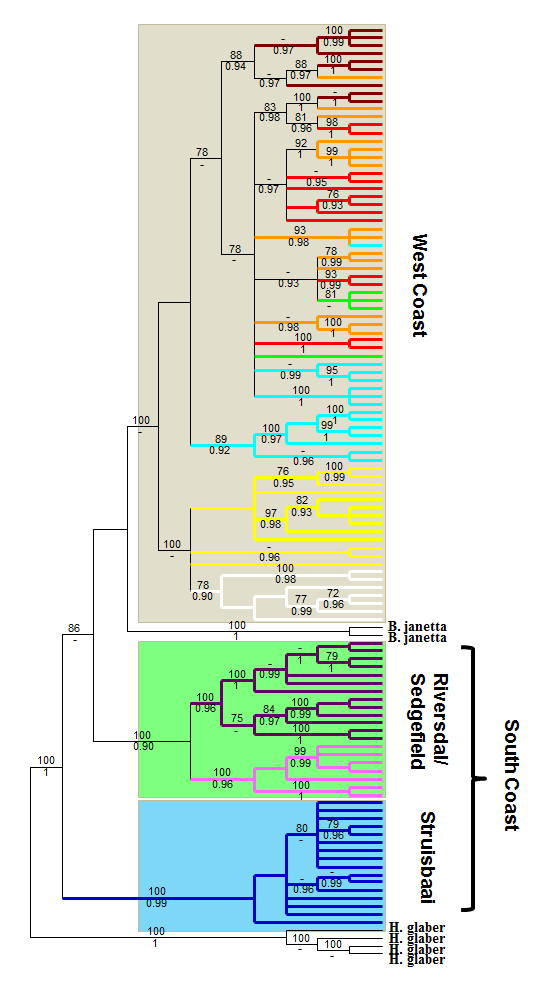

Supplement: Figure S1 — Bayesian phylogram demonstrating the different mitochondrial DNA clades across the sampled distribution. Bayesian phylogram obtained from the analyses based on combined cytochrome b and control region sequences demonstrating the different mitochondrial DNA clades detected in B. suillus from localities across the Cape Floristic Region. Values above each node represent posterior probabilities (Pp) derived from the Bayesian inference (MrBayes and BEAST) analyses and those below nodes are the Maximum Parsimony values. (TIF) [file pone.0107226.s001.tif]

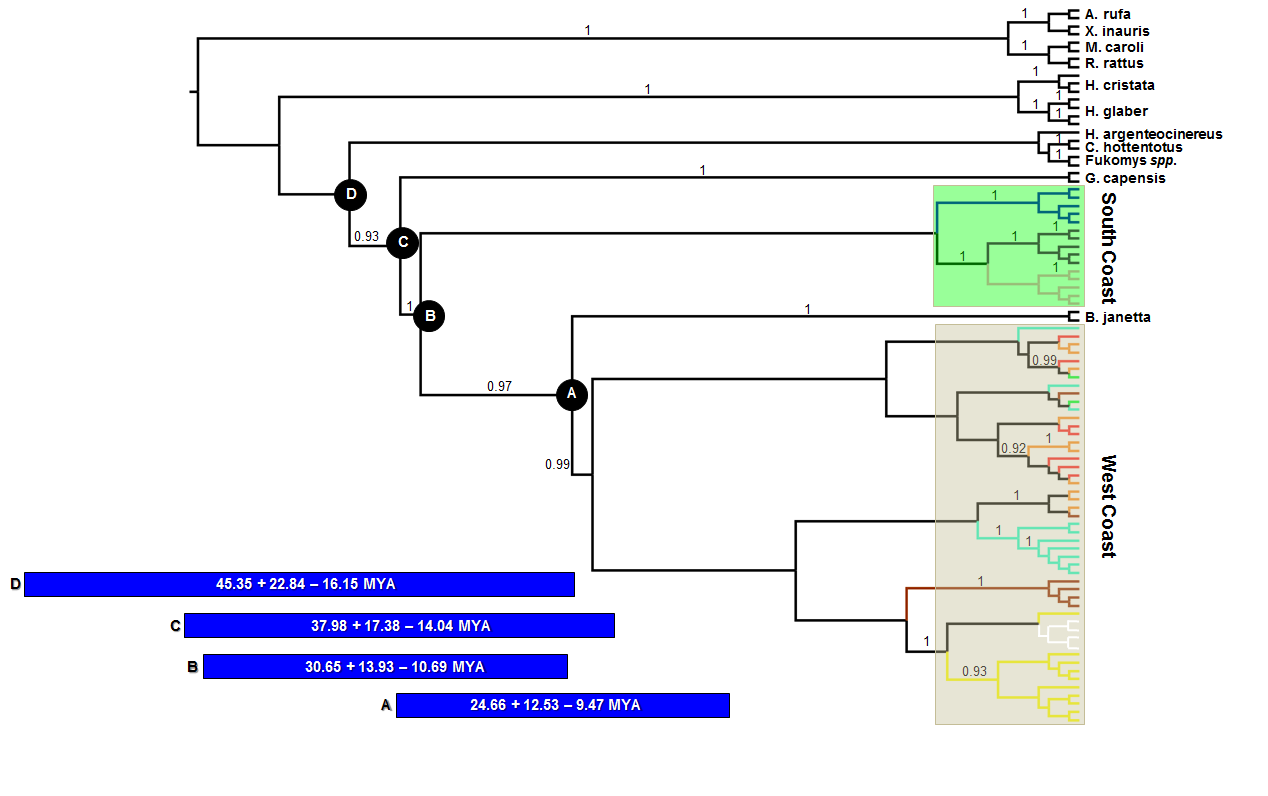

Supplement: Figure S2 — Bayesian phylogram from the BEAST analysis indicating the divergence dates between clades. Bayesian phylogram obtained from the BEAST analyses of the cytochrome b haplotypes among the 10 B. suillus sample sites across the Cape Florisitc Region. The values above each node represent the posterior probability (pP) values derived from the Bayesian inference analyses. The populations comprising the two clades evident across the distribution are shown. The divergence dates for four nodes (A–D) are indicated as bars which include the span of the divergence estimate for that particular node. (TIF) [file pone.0107226.s002.tif]
