# Supplementary material for: Local and Regional Scale Genetic Variation in the Cape Dune Mole-Rat, Bathyergus suillus
Source: PLoS One. 2014 Sep 17;9(9):e107226. doi: 10.1371/journal.pone.0107226 (PMC4167993; doi:10.1371/journal.pone.0107226)
Supplement: Table S1 — Summary information for the microsatellite loci used in this study. Locus summary information showing the estimated proportion of null alleles (Null), percentage missing data of the total 356 alleles/locus, proportion of missing alleles/locus, the genotyping error per genotype and FIS values for each of the microsatellite loci used in this study. (DOCX) [file pone.0107226.s003.docx]

**Table S1. Summary information for the microsatellite loci used in this study.**

| **Marker** | **Null** | **% Missing** | **Genotyping error** | **F_IS_** |
| --- | --- | --- | --- | --- |
| DMR1 | 0.20 | 7.3 | 0.002 | 0.41 |
| DMR5 | 0.04 | 5.6 | 0.002 | 0.11 |
| DMR7 | 0.03 | 10.6 | 0.007 | 0.018 |
| CH1 | 0.00 | 1.1 | 0.000 | -0.06 |
| Bsuil01 | 0.08 | 3.3 | 0.000 | 0.23 |
| Bsuil02 | 0.04 | 3.3 | 0.000 | 0.02 |
| Bsuil04 | 0.14 | 8.4 | 0.004 | 0.23 |
| Bsuil05 | 0.04 | 5.6 | 0.001 | 0.05 |
| Bsuil06 | 0.02 | 1.7 | 0.000 | -0.01 |

Table S1. Locus summary information showing the estimated proportion of null alleles (Null), percentage missing data of the total 356 alleles/locus , proportion of missing alleles/ locus, the genotyping error per genotype and F_IS_ values for each of the microsatellite loci used in this study.
