# Supplementary material for: Local and Regional Scale Genetic Variation in the Cape Dune Mole-Rat, Bathyergus suillus
Source: PLoS One. 2014 Sep 17;9(9):e107226. doi: 10.1371/journal.pone.0107226 (PMC4167993; doi:10.1371/journal.pone.0107226)
Supplement: Table S2 — Summary of loci which did not conform to Hardy-Weinberg Equilibrium in each population. Summary of loci which did not conform to Hardy-Weinberg Equilibrium (marked by an “x”) in each sampled population of B. suillus. (DOCX) [file pone.0107226.s004.docx]

**Table S2. Summary of loci which did not conform to Hardy-Weinberg Equilibrium in each population.**

| **Population** | **DMR1** | **DMR5** | **DMR7** | **CH1** | **Bsuil01** | **Bsuil02** | **Bsuil04** | **Bsuil05** | **Bsuil06** |
| --- | --- | --- | --- | --- | --- | --- | --- | --- | --- |
| Redelinghuys | x |  |  |  | x |  | x |  |  |
| Dwarskersbos | x |  |  |  |  |  | x |  | x |
| Sterkfontein | x |  |  |  |  |  |  | x |  |
| Piketberg |  |  |  |  | x |  | x |  |  |
| Vredenburg | x |  | x |  |  |  |  |  |  |
| Cape Town | x |  |  | x |  |  |  |  |  |
| Stanford |  | x |  |  | x |  | x |  |  |
| Struisbaai |  |  |  |  |  |  | x |  |  |
| Riversdal | x |  |  |  |  |  | x |  |  |
| Sedgefield |  |  |  |  | x |  |  |  |  |

Table S2. Summary of loci which did not conform to Hardy-Weinberg Equilibrium (marked by an “x” in the table) in each sampled population of *B. suillus*.
